# Cycloelatanene A and B: absolute configuration determination and structural revision by the crystalline sponge method†
†Electronic supplementary information (ESI) available. CCDC 1487142 and 1487143. For ESI and crystallographic data in CIF or other electronic format see DOI: 10.1039/c6sc04288k


**DOI:** 10.1039/c6sc04288k

**Published:** 2016-10-27

**Authors:** Shoukou Lee, Manabu Hoshino, Makoto Fujita, Sylvia Urban

**Affiliations:** a Department of Applied Chemistry , School of Engineering , The University of Tokyo , Hongo, Bunkyo-ku , Tokyo , 113-8656 , Japan . Email: mfujita@appchem.t.u-tokyo.ac.jp; b School of Science (Discipline of Applied Chemistry and Environmental Science) , RMIT University , GPO Box 2476 , Melbourne 3001 , Victoria , Australia . Email: sylvia.urban@rmit.edu.au

## Abstract

Cycloelatanene A and B are marine natural products first reported a few years ago. Their relative structures had been elucidated by an extensive NMR study and found to be epimers. However, their absolute configurations had not been established because they were isolated in only minute quantities as oily compounds. In this study, the complete structures of cycloelatanene A and B, including absolute configurations, were determined by the crystalline sponge method. The structure analysis confirmed the unique tricyclic structure involving a spiro[5.5]undecene skeleton. One stereogenic centre at C4 was revised as a result of this analysis. Since it only took 1–2 weeks to complete the experiments using the crystalline sponge method (guest-soaking followed by crystallographic analysis), this method is now highly recommended as a first port of call to achieve complete natural product structure elucidation.

## Abstract 2

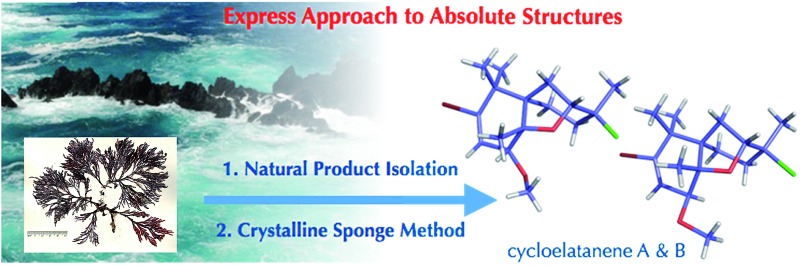
The crystalline sponge method revealed the absolute configuration of cycloelatanene A and B.

## Introduction

Cycloelatanenes A and B (**1** and **2**, respectively) are C16 chamigrenes which were first reported from the Australian marine red alga, *Laurencia elata* in 2011.^1^ Their structures were deduced *via* detailed spectroscopic methods and the relative configuration was established using selective 1D NOE NMR experiments. The compounds have a unique tricyclic scaffold involving a spiro[5.5]undecene structure and an ether oxygen atom that bridges the two C6 rings in the spiro structure. Whilst their relative structures had been proposed,^1^ the absolute configurations of the compounds have remained undetermined. Although X-ray crystallography is the most convincing method to determine the absolute configuration,^2,3^ the compounds are not amenable to common X-ray analysis because they only occur as oily compounds in minute quantities.

**Fig. 1:**
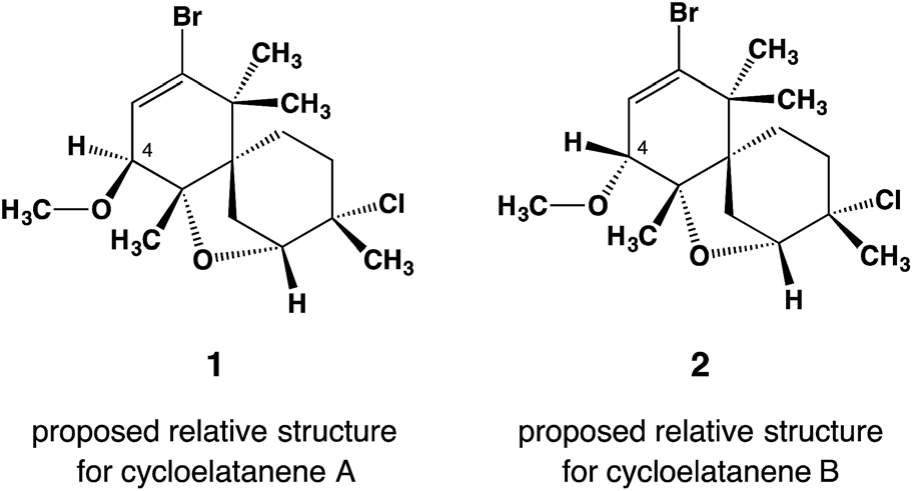
Reported relative structures of cycloelatanene A (**1**) and B (**2**) as elucidated by a detailed NMR study. The compounds are epimers with opposite configuration at C4. Note that these structures have been revised as shown in Fig. 2.

The crystalline sponge method is a recently developed X-ray technique that can analyze the structure of scarcely available oily compounds by absorbing the guest into the ordered cavities of crystalline porous complexes (crystalline sponges).^4^ The absolute structure of the absorbed guest can be determined even if the guest does not contain heavy atoms because anomalous scattering can be observed from the host heavy atoms.^5^ This great advantage has recently been applied to the absolute structure determination of natural products, where no other means except for the crystalline sponge method could directly address the absolute configuration of the compounds.^6^ The present study reports on the absolute configuration determination of cycloelatanene A and B *via* the crystalline sponge method. This methodology has not only been able to confirm the unique tricylic framework of the cycloelatanenes, which possess a spirocarbon center, but has also led to the revision of one of the chiral centers in these structures (Fig. 1).

## Results and discussion

[(ZnI_2_)_3_(tpt)_2_(cyclohexane)_*x*_] (**3**, tpt = 2,4,6-tris-(4-pyridyl)-1,3,5-triazine)^7^ was used as the crystalline sponge in this study. A single crystal of **3** (typically approx. 200 × 150 × 100 μm^3^) was placed in a vial and immersed in a small amount of cyclohexane (45 μL). For cycloelatanene B (**2**), five replicate vials were prepared and a 1,2-dichloroethane solution (5 μL) of **2** (1 μg μL^–1^, 5 μg) was added to each of them. The vials were equipped with a syringe needle for slow solvent evaporation. Guest soaking was carried out at 50 °C for 1 d. After confirming solvent evaporation, all the guest-absorbed crystals were subjected to a short exposure time sample screening with an in-house X-ray diffractometer. One crystal was selected that produced the best diffraction pattern giving sharp and non-split spots and the highest resolution. The single-crystal X-ray diffraction experiment was performed on an in-house X-ray diffractometer with Mo Kα radiation.

**Fig. 2:**
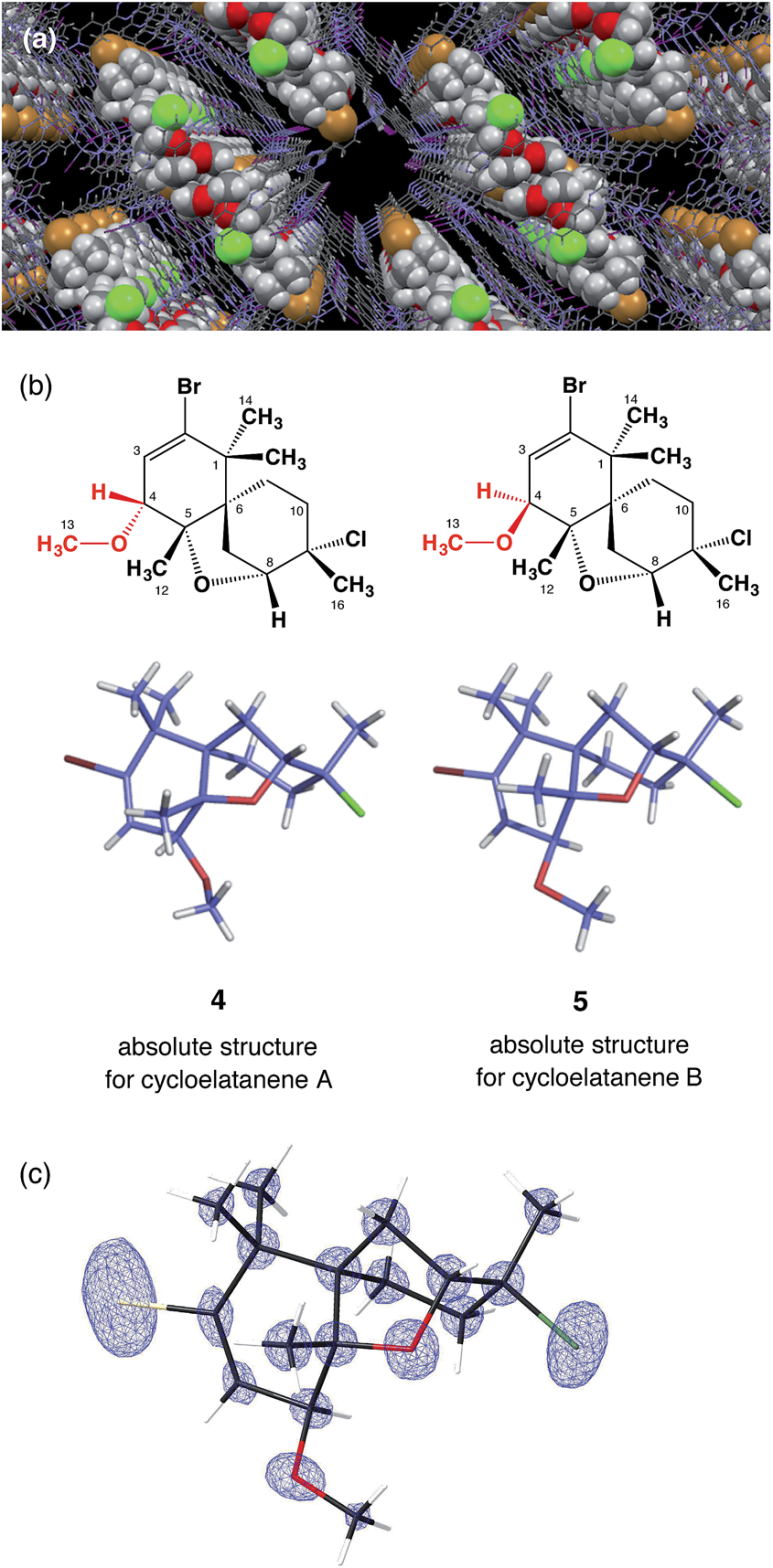
Crystal structures of cycloelatanene A (**4**) and B (**5**). (a) Guest **5** (space filling presentation) embedded in the pore of crystalline sponge **3** (wire frame representation). (b) Crystal structures of **4** and **5** in stick representation. (c) *F*_o_ electron density map for **5** superimposed on its wireframe representation.

Due to the inclusion of the chiral guest, the net host–guest structure becomes chiral and the inherent centrosymmetric *C*2/*c* space group of host **3** turns into the non-centrosymmetric *C*2 space group, as confirmed based on the extinction rule systematic absence of reflections. The crystallographic analysis revealed two independent guest molecules per asymmetric unit with estimated populations of 71.0% and 14.5% (Fig. 2). The Flack parameter calculated by the Parsons' method^8^ was 0.033(5) and we were able to determine the absolute configuration of **2**, which could not be addressed by the previous NMR studies. The data quality obtained structure model reliably confirmed the unique tricyclic skeleton. The electron density map is clear (Fig. 2c) and refinement was achieved without applying any restraints on the framework. Guest molecules were refined with restraint on atomic displacement parameters (SIMU and RIGU). The five chiral centers in the skeleton were determined to be 4*R*, 5*S*, 6*R*, 8*R*, 9*S*. As a result of this analysis, the stereochemical assignment at the C4 stereogenic center was revised. The revised structure of cycloelatanene B (newly denoted as **5**) has the same relative stereochemistry as structure **1** on the methoxy group at C4 (Fig. 2b). The sample was recovered from the vials and the ^1^H NMR of the compound was acquired and compared to the natural product before the soaking procedure. No changes in the chemical shifts for the H4 or 13-OCH_3_ signals were evident (Fig. S3†), thus eliminating the possibility of epimerization having occurred at the C4 stereogenic center during the soaking experiments.

According to the previous NMR studies, cycloelatanene A and B are epimers with opposite configurations at C4. Thus the structure of cycloelatanene A should also be revised as **4** (as denoted in Fig. 2b). To conclusively determine the structure of cycloelatanene A, the compound was also subjected to structure analysis by the crystalline sponge method. After some trial-and-error experiments, the soaking conditions were optimized and the diffraction study was performed with the best-diffracted single-crystal on an in-house X-ray diffractometer; for this compound, Cu Kα radiation gave better quality data than Mo Kα radiation.

In the crystallographic analysis, one guest molecule per asymmetric unit was clearly observed. As expected, cycloelatanene A (**4**) was shown to have an inverted methoxy group at the C4 center (Fig. 2b). The Flack parameter calculated by the Parsons' method was 0.264(9). The high Flack value is presumably due to insufficient guest occupancy (∼47%) at the binding site of the crystalline sponge. Despite the Flack parameter value, the absolute configuration of **4** is suggested to be 4*S*, 5*S*, 6*R*, 8*R*, 9*S* by analogy with the absolute configuration deduced for its C4-epimer, **5**, based on the similar optical rotations between **4** and **5**.^1^

**Fig. 3:**
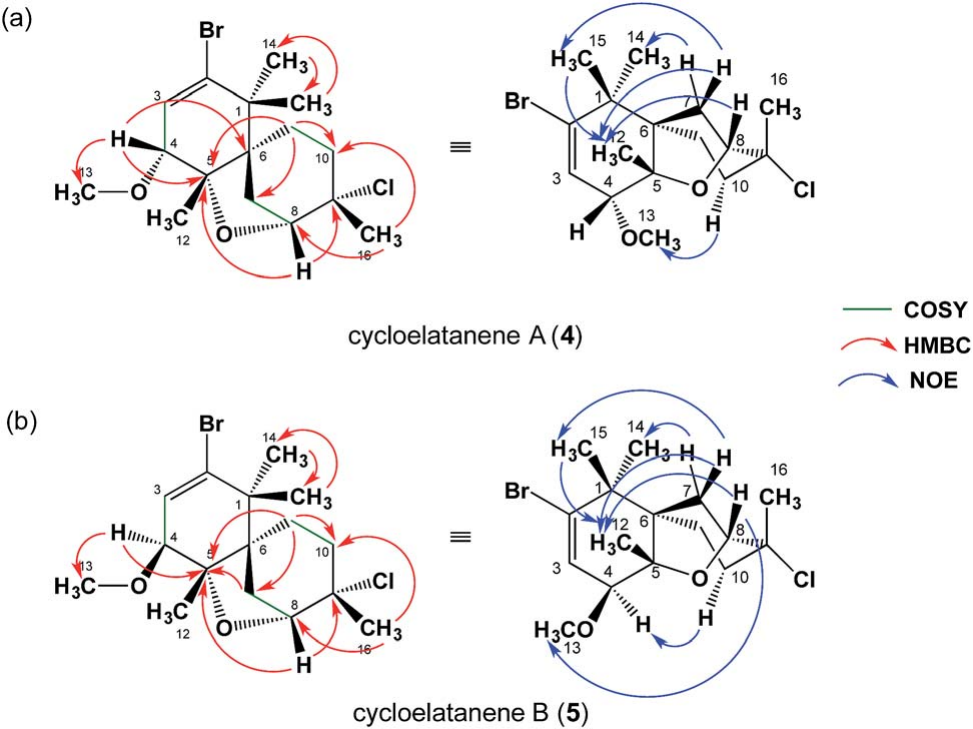
Structural elucidation based on the NMR study. ^1^H–^1^H COSY, key HMBC, and key NOE correlations in (a) **4** and (b) **5** are indicated. For more details, see supporting information and Fig. S2.†

As a result of the revision of the C4 stereogenic center, as deduced by the crystalline sponge method, the 1D NOE NMR spectra acquired for cycloelatanenes A (**4**) and B (**5**) were reanalyzed. The revised data are given in Fig. 3 (see also Fig. S2†) and in Tables S1 and S2,† respectively. A number of additional weak NOE correlations in both cycloelatanenes had also been observed and due to the close nature of some of the chemical shift assignments in both compounds, it is highly likely that both sets of protons were inadvertently irradiated (*e.g. δ* 1.68/1.62 and 3.63/3.67 in **4** and 1.68/1.70 in **5**). This meant that some NOEs reported in the original study required revision and reassignment. In addition, based on the NOE data, the proton signals assigned to H10a and H10b in the original report of the structures of the cycloelatanenes^1^ should be reversed.

After close re-examination of the data, key NOE enhancements placed 12-CH_3_, 14-CH_3_, 15-CH_3,_ H7ab, H8, H10a and H11ab at the convex of the rigid and bent tricyclic framework in both **4** and **5**. The two epimers **4** and **5** could be discriminated by an additional key NOE enhancement observed between the two substituents placed in close proximity in the concave region of the framework: *i.e.*, H10b with 13-OCH_3_ in **4** and H10b with H4 in **5** in the concave region of the molecules (see Fig. S2†). On this basis the C4 stereogenic center could be deduced for both cycloelatanenes A and B and was consistent with the findings obtained from the crystalline sponge method.

**Fig. 4:**
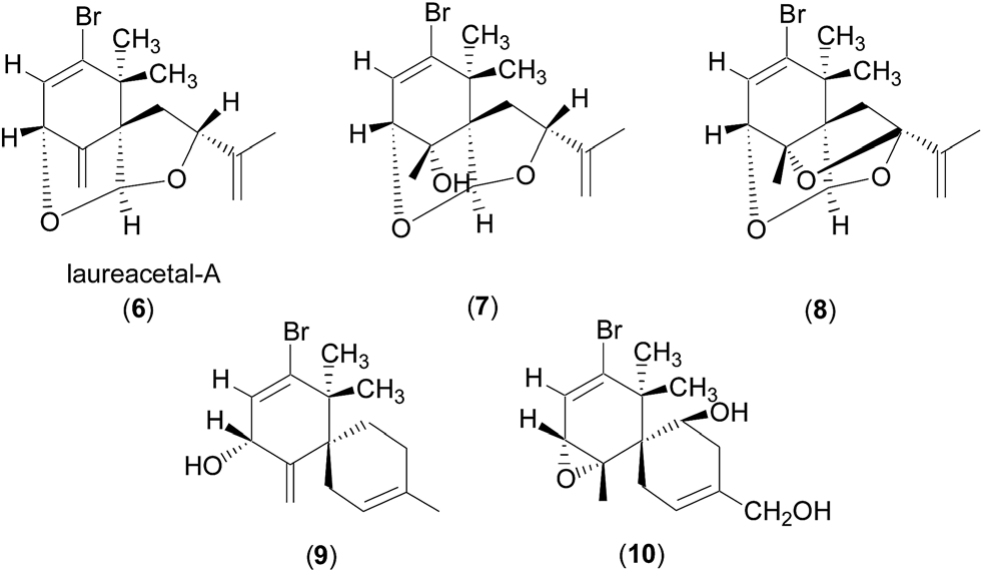
Structures of **6–10**.

Further support for the revised assignment of the C4 stereogenic center in the cycloelatanenes was based on analysis of the coupling constants between the olefinic H3 and the methine H4. The crystal structures reveal that the dihedral angles *ψ*_H3–C3–C4–H4_ are 63° and 83° for **4** and **5**, respectively (Fig. 4).

The observed *J*_H3–H4_ values of 6.0 and 1.6 Hz for **4** and **5**, respectively, are thus consistent with the crystal structures as the *J* value decreases with increasing *ψ* value in the range of 0° < *ψ* < 90° (Karplus equation).^9^ The *J*_H3–H4_ value for **4** (6.0 Hz) is also consistent with the values observed for structurally related compounds **6–10**, which showed *J* values of 4–7 Hz (ref. 10–12) with the proton facing up at the stereogenic centre of interest (C4 in **4**). Laureacetal A (**6**) has had its absolute configuration confirmed.^13^

## Conclusions

In conclusion, the crystalline sponge method has been used to unequivocally deduce the absolute configuration of cycloelatanenes A and B, which possess five chiral centers. While initial NMR spectroscopy was successful in elucidating the gross structures and the relative configuration for four of the five chiral centers, the sponge method has provided confirmation of the absolute configuration, resulting in one chiral center being revised. Reanalysis of the 1D NOE NMR experiments in combination with a comparison of the coupling constants for structurally related natural products also supports the final stereogenic center reassignment.

The crystalline sponge method is a revolution in complete structure determination, particularly in the area of natural products chemistry. Its ability to establish the absolute configuration of highly complex bioactive natural products is extraordinary and is certain to pave the way for the re-analysis of many synthetic or natural compounds, especially in the field of drug discovery. Based on these findings, it is recommended that structure determination of new chiral entities be based on combining NMR spectroscopy and mass spectrometry with the crystalline sponge method where possible. Whilst limitations of this approach include the necessity for expertise in sponge methodology and crystallography and the fact that not all molecules will be absorbed by the sponge method, this complete structure elucidation package approach, where achievable, is remarkable.

## References

[cit1] (b) *Laurencia elata* has been transferred to a new genus and is now known as *Coronaphycus elatus* (*C. Agardh*) Metti: Metti Y. Millar A. J. K. Steinberg P., J. Phycol., 2015, 51 , 929 –942 . 26986889 10.1111/jpy.12333

[cit2] Flack H. D., Bernardinelli G. (2008). Chirality.

[cit3] Nicolaou K. C., Snyder S. A. (2005). Angew. Chem., Int. Ed..

[cit4] Inokuma Y., Yoshioka S., Ariyoshi J., Arai T., Hitora Y., Takada K., Matsunaga S., Rissanen K., Fujita M. (2013). Nature.

[cit5] Yoshioka S., Inokuma Y., Hoshino M., Sato T., Fujita M. (2015). Chem. Sci..

[cit6] Urban S., Brkljaca R., Hoshino M., Lee S., Fujita M. (2016). Angew. Chem., Int. Ed..

[cit7] Biradha K., Fujita M. (2002). Angew. Chem., Int. Ed..

[cit8] Parsons S., Flack H. D., Wagner T. (2013). Acta Crystallogr., Sect. B: Struct. Sci., Cryst. Eng. Mater..

[cit9] Karplus M. (1963). J. Am. Chem. Soc..

[cit10] Suzuki T., Kurosawa E. (1979). Chem. Lett..

[cit11] Suzuki M., Segawa M., Suzuki T., Kurosawa E. (1985). Bull. Chem. Soc. Jpn..

[cit12] Kurata K., Suzuki T., Suzuki M., Suzuki M., Kurosawa E., Furusaki A., Suehiro K., Matsumoto T., Katayama C. (1983). Chem. Lett..

[cit13] Suzuki T., Furusaki A., Hashiba N., Kurosawa E. (1977). Tetrahedron Lett..

