# Supplementary material for: Cycloelatanene A and B: absolute configuration determination and structural revision by the crystalline sponge method
Source: Chem Sci. 2016 Oct 27;8(2):1547–50. doi: 10.1039/c6sc04288k (PMC5452270; doi:10.1039/c6sc04288k)
Supplement: Supplementary file 1 [file SC-008-C6SC04288K-s001.pdf]

Electronic Supplementary Material (ESI) for Chemical Science.
This journal is © The Royal Society of Chemistry 2016

Supporting Information for

Cycloelatanene A and B: Absolute Configuration Determination and Structural Revision by the NMR-Coupled Crystalline Sponge Method

Shoukou Lee,[1] Manabu Hoshino,[1] Makoto Fujita,*[1] Sylvia Urban*[2]

[1]Department of Applied Chemistry, School of Engineering, The University of Tokyo.
[2]School of Science (Discipline of Chemistry), RMIT University.
E-mail: sylvia.urban@rmit.edu.au, mfujita@appchem.t.u-tokyo.ac.jp

Table of Contents

**Reagents and Equipment**	**S2**
**X-ray Diffraction Analysis**	**S2-S5**
**NMR Analysis**	**S5-S11**
**References**	**S11**

Reagents and Equipment

Solvents and reagents were purchased from TCI Co., Ltd., WAKO pure Chemical Industries Ltd., and Sigma-Aldrich Co and used without further purification. Cycloelatanene A and B were isolated by the Urban group.[1] The crystalline sponge $[(ZnI_2)_3(tpt)_2•(cyclohexane)_x]$ (**3**, tpt = 2,4,6-tris(4-pyridyl)-1,3,5-triazine) was prepared according to the reported procedure.[2] A screw-top microvial (Osaka chemical, cat. No. 11090620), screw cap with septum seal (Osaka chemical, cat. No. 53951-09FB) and a syringe needle (TERUMO, cat. No. NN-2116R) were used for guest inclusion into the porous crystals.

X-ray Diffraction Analysis

Structural analysis of cycloelatanene A (4) by crystalline sponge method

A single crystal of porous complex $[(ZnI_2)_3(tpt)_2•(cyclohexane)_x]$ (**3**), immersed in cyclohexane (45 µL) in a small vial, was treated with 1,2-dichloroethane solution (5 µL) of **4** (1 µg µL^{-1}, 5 µg) for 3 d at 50 °C without solvent evaporation then 1 d at 50 °C with solvent evaporation through a needle for the uptake of **4** into the pores of **3**. The prepared red single crystal of **3•4** complex with a dimension of 254 x 139 x 68 µm^3 was used for the X-ray diffraction data collection. A single crystal X-ray diffractometer (SuperNova by Rigaku Oxford Diffraction) equipped with a fine-focused Cu *Kα* X-ray source (Nova) and a high-sensitivity CCD detector (Altas S2 CCD detector) was used for the diffraction data collection. The sample crystal was cooled to 100 K using a cold nitrogen stream (Cobra by Oxford Cryosystems). Two different detector positions (2θ = −41.46° or 112.00°) were applied for separately collecting diffraction data at the high-angle and low-angle regions. Exposure time for each diffraction image par 1° oscillation in the ω scan was selected to fulfill the suitable I/σ value; 172.28 sec to fulfill $I/\sigma > 6.75$ for the high-angle region and 33.64 sec to fulfill $I/\sigma > 15.00$ for the low-angle region. The sample-to-detector distance was 53 mm. On this setting, the maximum data resolution (d) is ca. 0.80 Å. The complete data collection took 70 h.

Collected data were processed using the program CrysAlisPro ver. 1.171.38.41.[3] In this process, numerical absorption correction based on Gaussian integration over a multifaceted crystal model was applied to correct significant Cu *Kα* X-ray absorption by the sample crystal. Empirical absorption correction using spherical harmonics was also applied. All crystal structures were solved using the program SHELXT ver. 2014/5[4] and refined using the program SHELXL ver. 2014/7[5] as mentioned above.

*Crystallographic data of **3•4** complex*: $C_{114.70}H_{129.68}Br_{0.47}Cl_{3.14}I_{12}N_{24}O_{0.93}Zn_6$, M = 3922.89, monoclinic *C*2, a = 35.1845(11) Å, b = 14.8561(2) Å, c = 31.0855(8) Å, β = 101.739(3)°, V = 15908.7(7) $Å^3$, Z = 4, GoF = 1.021, R_1 = 0.0542, wR_2 = 0.1894, Flack parameter calculated by Parsons' method; 0.264(9). CCDC deposit number 1487142. The CCDC data can be obtained free of charge from the Cambridge Crystallographic Data Centre (http://www.ccdc.cam.ac.uk/data_request/cif).

All non-hydrogen atoms in the framework were refined without using restraint and constraint. The guest molecule was refined with restraint on atomic displacement parameters (SIMU) and DFIX based on the X-ray diffraction data of **4**. The Flack parameter calculated by Parsons' method, 0.264(9), was not sufficient to discuss the absolute configuration. This was probably because of the low occupancy of **4**; about 47% of the pores were unoccupied and remained as centrosymmetric. After checkCIF (http://checkcif.iucr.org/), the alert of low bond precision on C-C bonds appeared. This was also because of the low occupancy of the guest molecule and severely disordered solvent compounds in the pores. Thus, the geometries in a disordered part (especially 1,2-dichloroethane molecules) were qualitative.

Structural analysis of cycloelatanene B (5) by crystalline sponge method

A single crystal of porous complex **3**, immersed in cyclohexane (45 μL) in a small vial, was treated with 1,2-dichloroethane solution (5 μL) of **5** (1 μg μL^{-1}, 5 μg) for 1 d at 50 °C for the uptake of **5** into the pores of **3**. The prepared single crystal of **3•5** complex with a dimension of 350 x 200 x 120 μm^3 was used for the X-ray diffraction data collection. A single crystal X-ray diffractometer (Rigaku XtaLAB P200) equipped with a rotating anode X-ray source (Mo *K*α radiation) and a hybrid photon counting detector (DECTRIS Pilatus 200K) was used for the diffraction data collection. The sample crystal was cooled to 93 K using a cold nitrogen stream. Exposure time was 18.0 sec, the number of redundancy was 5.0 and the width was 0.50˚. The maximum data resolution (*d*) is ca. 0.83 Å. The complete data collection took 9 h. Collected data were integrated, corrected, and scaled using the program CrysAlisPro ver 1.171.38.41.[3] Empirical absorption correction was applied in this process.

All crystal structures were solved using the program SHELXT ver. 2014/5[4] and refined using the program SHELXL ver. 2014/7.[5] All non-hydrogen atoms were refined anisotropically. All hydrogen atoms are generated using the proper HFIX command and refined isotropically using the riding model. Solvent cyclohexane molecules suggested

in the electron density map were refined using some restraints (DFIX, DANG, SIMU, and ISOR) because corresponding electron densities were obscured due to their thermal motion and disordering. Short intermolecular interactions were found by the HTAB command.

*Crystallographic data of **3•5** complex*: $C_{118.68}H_{134.52}Br_{0.86}Cl_{0.86}I_{12}N_{24}O_{1.71}Zn_6$, M = 3938.17, monoclinic *C*2, a = 35.2593(5) Å, b = 14.8434(1) Å, c = 31.0697(4) Å, β = 101.611(1)°, V = 15928.1(3) $Å^3$, Z = 4, GoF = 1.037, R_1 = 0.0340, wR_2 = 0.0997, Flack parameter calculated by Parsons' method; 0.033(5). CCDC deposit number 1487143. The CCDC data can be obtained free of charge from the Cambridge Crystallographic Data Centre (http://www.ccdc.cam.ac.uk/data_request/cif).

All non-hydrogen atoms in the framework were refined without using restraint and constraint. Guest molecules were refined with restraint on atomic displacement parameters (SIMU and RIGU).

The populations of observed two independent guest molecules per an asymmetric unit were estimated to be 71.0% (**5A**), 14.5% (**5B**) respectively according to the structure refinement. Thus the guest-absorbed complex can be formulated as $[(ZnI_2)_3(tpt)_2(\mathbf{5})_{0.43}(cyclohexane)_{5.5}]$.

(a)

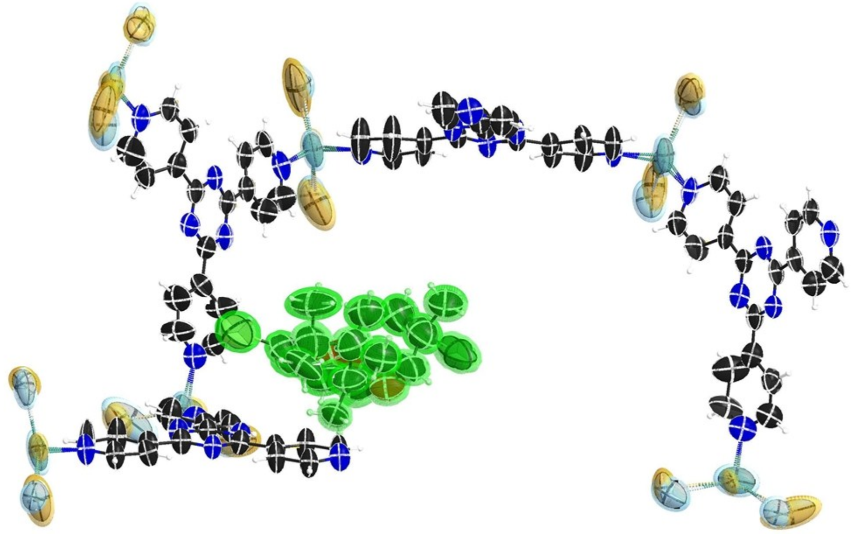

(b)

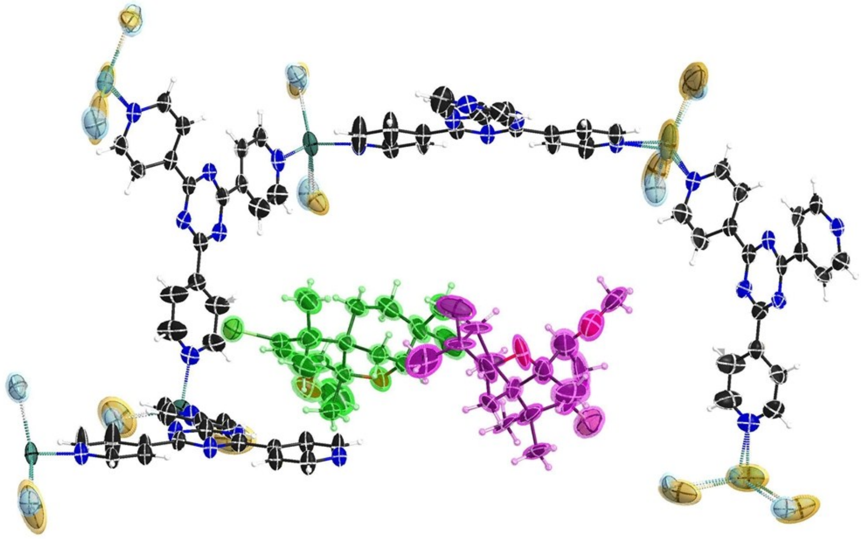

Figure S1. ORTEP diagram with 50% probability in the asymmetric unit of (a) **3•4** and (b) **3•5**. Enclosed atoms by the PART command in SHELXL were represented using the difference color code. Solvent molecules co-enclathrated are omitted for clarity.

NMR Analysis

Updated NMR analysis of cycloelatanene A (4)

Table 1. ^{1}H (500 MHz) and ^{13}C (125 MHz) NMR of cycloelatanene A (**4**) in $CDCl_3$.

Pos	δ_H (*J* in Hz)	δc^a, mult	gCOSY	gHMBC	1D NOE
1		45.4 s			
2		139.1 s			
3	6.21 d (6.0)	128.2 d	4	1, 2, 5	4, 14
4	3.67 dd (6.0, 0.5)	82.4 d	3	2, 3, 5, 6, 12, 13	12 (w)
5		85.7 s			
6		48.8 s			
7a	2.21 ddd (12.0, 7.5, 4.0)	33.4 t	7b, 8, 11a(w)	6, 8, 9, 11	7b, 8, 12, 15
7b	1.68 d (12.5)		7a	8, 9, 11	7a, 8, 13(w), 14
8	4.30 dd (7.5, 1.0)	84.3 d	7a	5, 6, 9, 10	7a, 7b, 12, 15, 16
9		74.7 s			
10a	1.83 dd (13.5, 7.0)	37.5 t	10b, 11b	6, 8, 9, 11, 16	10b, 11b(w), 16
10b	2.94 ddd (13.5, 13.0, 8.0)		10a, 11a, 11b	9, 11, 16	10a, 11a(w), 13
11a	1.98 m	31.6 t	7a, 10b, 11b	6, 7, 9, 10	11b, 15
11b	1.54 ddd (13.5, 12.5, 6.5)		10a, 10b, 11a, 16(w)	5, 6, 7, 10	11a, 14
12	1.29 s	30.1 q		4, 5, 6	
13	3.63 s	60.3 q		4	3, 4, 10b, 12
14	1.13 s	29.2 q		2, 15	13(w)
15	1.14 s	22.7 q		2, 14	12
16	1.62 s	28.1 q	11b(w)	8, 9, 10	8, 10a

[a] Carbon assignments based on HSQCAD and DEPT NMR experiments

w = weak correlation or enhancement

*Updated and revised table of assignment for cycloelatanene A (**4**).*

Revisions:

1. 10a and 10b assignments swapped in chemical shift column.
2. Consequently when comparing to original data published in *Phytochemistry* **2011**, *72*, 2081-2089 the 10a and 10b assignments have been swapped.
3. Single irradiation NOE NMR data was reviewed. In some cases additional NOE's (weak) were noted. In other cases a review of the NOE's indicated that 1.68/1.62 could have been irradiated together. This means the NOE from 1.68 to 1.62 has now been removed from the table above.

In the original table of NMR assignments the problematic NOE's were:

1. 1.14 to 13(w). This should have been 1.13 to 13(w).
2. 2.94 to 13. In reviewing other NOE's to 2.94 this NOE is actually correct and revises 2.94 which was previously assigned as H10a to H10b. The review of H10a and H10b also applies to cycloelatanene B.
3. Finally the other problem NOE was 4.30 to 3.63. In reviewing the single irradiation for 4.30, the signal at 3.63 could have been a spike (noise).
4. Additional NOE's were found from 3.67 to 12 (H4 to H12) and 1.83 to 16 (H10a to 16) as well as from 1.68 to 13 (7b to 13).

Upon revision of these NOE's the relative configuration of cycloelatanene A can be revised to have the OCH_3 substituent at position 4 as down and the H4 at position 4 to be up. This is consistent with the absolute configuration assignment obtained by the sponge crystalline method.

Updated NMR analysis of cycloelatanene B (5)

Table 2. ^{1}H (500 MHz) and ^{13}C (125 MHz) NMR of cycloelatanene B (**5**) in $CDCl_3$.

Pos.	δ_H (*J* in Hz)	δ_C[a], mult	gCOSY	gHMBC	1D NOE
1		45.1 s			
2		135.2 s			
3	5.98 d (1.6)	131.0 d	4	1, 2, 5	4, 12, 13
4	4.17 d (1.6)	80.3 d	3	2, 3, 5, 13	3, 10b, 7b, 12(w), 13
5		86.8 s			
6		50.1 s			
7a	2.22 ddd (12.0, 7.5, 4.0)		7b, 8,	5, 6, 9, 11	7b, 8, 12, 15
7b	1.68 d (12.0)	32.5 t	7a	5, 6, 9, 11	H4, 7a, 14
8	4.29 brd (7.5)	84.5 d	7a	5, 9, 10	7a, 7b, 12, 13(w), 16
9		72.7 s			
10a	1.95 dd (13.0, 6.0)		10b, 11b	6, 8, 9, 11,16	10b, 11a, 11b
10b	2.58 ddd (14.0, 13.5, 7.0)	38.2 t	10a, 11a, 11b, 16	9, 11, 16	4, 7b, 10a, 11a, 11b
11a	1.70 m		10b		7a, 8, 15
11b	1.56 ddd (14.5, 14.5, 6.0)	30.4 t	10a, 10b	5, 6, 7, 10	10a, 10b(w), 11a, 14
12	1.27 s	22.7q		4, 5, 6	3(w), 4(w), 7a, 8(w), 13, 15
13	3.53 s	58.2 q		4	3, 4, 12
14	1.09 s	28.6 q		1, 2, 6, 15	7b, 11b, 15
15	1.18 s	22.6 q		1, 2, 6, 14	7a, 11a(w), 12, 14
16	1.60 s	26.9 q	10a(w)	8, 9, 10	8, 10a

[a] Carbon assignments based on HSQCAD and DEPT NMR experiments

w = weak correlation or enhancement

*Updated and revised table of assignment for cycloelatanene B (**5**).*

Revisions:

1. 10a and 10b assignments have been swapped in chemical shift column (see note above).
2. Consequently when comparing to original data published in *Phytochemistry* **2011**, *72*, 2081-2089 the 10a and 10b assignments have been swapped.
3. Single irradiation NOE NMR data was reviewed. In some cases additional NOE's (weak) were noted. In other cases a review of the NOE's indicated that 1.68/1.70 would have been irradiated together. The NOE's have been reviewed and updated in light of this.

In original table assignment the problematic NOE's were:

1. 4.17 to 11a. This should have been 4.17 to 1.68 or 7b.
2. 2.22 to 11a or 1.70. This should have been to H7b only so the 11a NOE has been removed.
3. There was an additional NOE from 1.68 to H4. The problem here is that 7a and 11a were irradiated together so this has now been reassigned.
4. An additional weak NOE was seen from 4.29 to 13.
5. An NOE from 2.58 to 7b has been added.
6. The NOE from 1.56 to 3.53 (13) was removed (this was a spike only observed in one of the traces).
7. There was an additional NOE evident from 1.27 to 13.
8. There was an additional NOE evident from 3.53 to 12.

Upon revision of these NOE's the relative configuration of cycloelatanene A can be revised to have the OCH_3 substituent at position 4 as up and the H4 at position 4 to be down. This is consistent with the absolute configuration assignment obtained by the sponge crystalline method.

Key NOEs

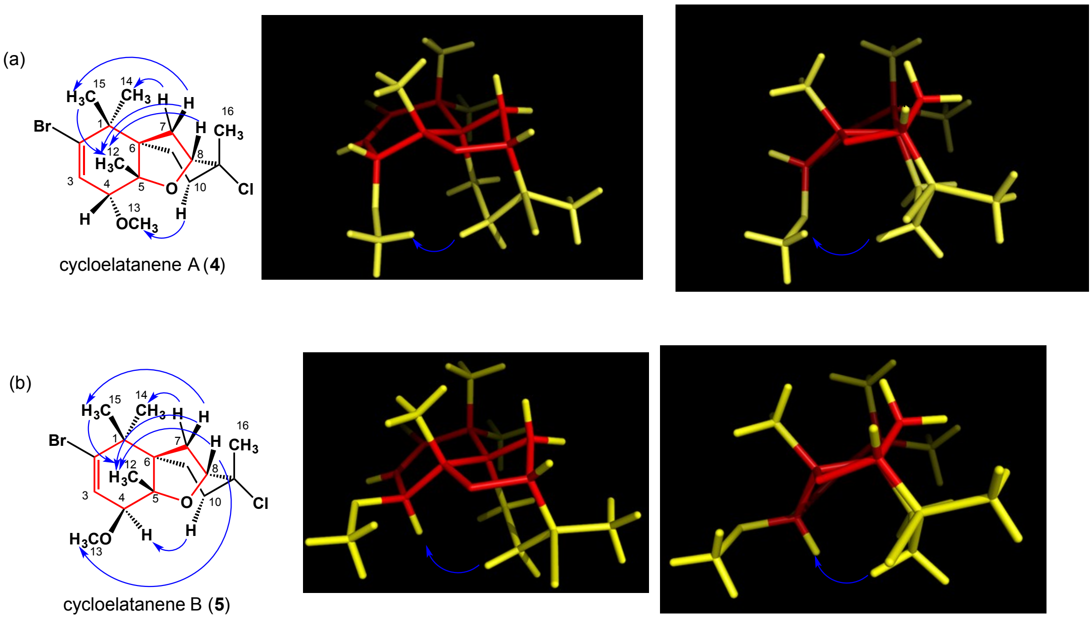

Figure S2 Key NOEs in (a) cycloelatanene A (**4**) and (b) cycloelatanene B (**5**).

^{1}H NMR analysis of recovered cycloelatanene B (5)

The ^{1}H NMR spectrum was recorded on a JEOL (500 MHz) NMR spectrometer and collected at 300 K. The chemical shifts are reported in parts per million (ppm) relative to an internal standard tetramethylsilane (δ = 0.00 ppm) for $CDCl_3$.

Following the X-ray crystal structure analysis, all 5 vials containing less than 25 μg of **5** were washed with small portions of fresh dichloromethane. The solutions were collected into one vial and concentrated in vacuo. The residue was dissolved in $CDCl_3$ and placed in an NMR tube.

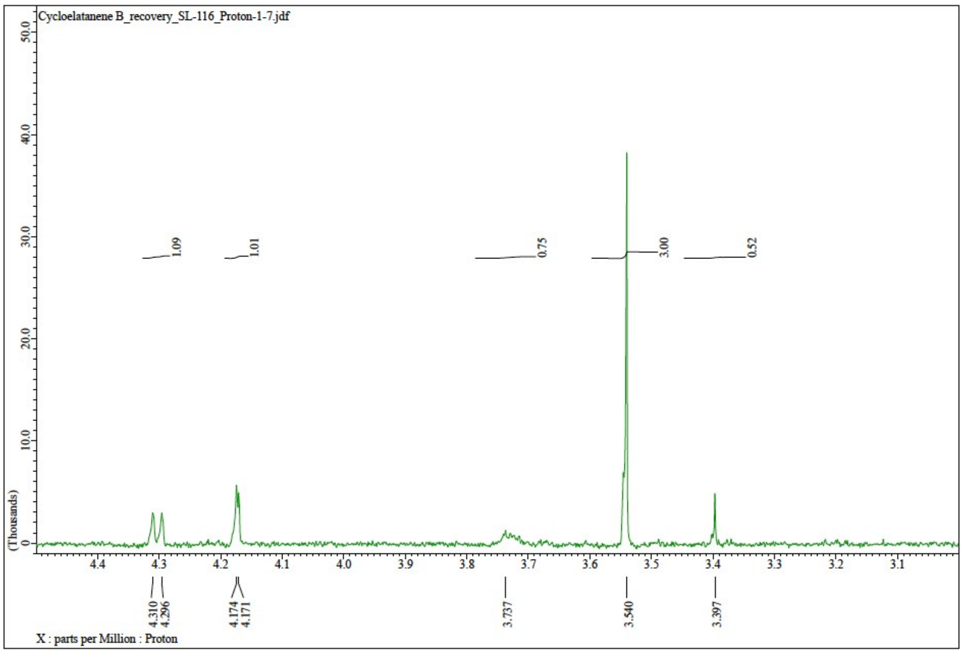

Figure S3. Expansion of the ^{1}H NMR spectrum of cycloelatanene B (**5**) recovered from the vials used in the soaking experiment at a range of 3.0 ~ 4.5 ppm. The H4 and the methoxy signal of cycloelatanene B were observed at δ 4.17 and 3.54, respectively (consistent with the chemical shifts observed before the soaking procedure). Cycloelatanene A shows these protons at δ 3.67 and 3.63 respectively. That these chemical shifts were absent after the sample recovery eliminated the possibility of epimerization occurring during the soaking experiment.

References

1 Dias, D. A.; Urban, S. Phytochemical studies of the southern Australian marine alga, *Laurencia elata*. *Phytochemistry* **2011**, *72*, 2081–2089.

2 Biradha, K., Fujita, M. A Spring-like 3D-Coordination Network that Shrinks or Swells in Crystal-to-Crystal Manner upon Guest Removal or Readsorption. *Angew. Chem. Int. Ed.* **2002**, *41*, 3392–3395.

3 CrysAlisPro, Agilent Technologies, Version 1.171.38.41 (release 28-09-2015 CrysAlis171 .NET).

4 Sheldrick, G. M. *SHELXT* – Integrated space-group and crystal-structure determination *Acta Crystallogr. Sect. A* **2015**, *71*, 3–8.

5 Sheldrick, G. M. Crystal structure refinement with SHELXL. *Acta Crystallogr. Sect. C* **2015**, *71*, 3-8.
